# The Functional Role of CONNEXIN 26 Mutation in Nonsyndromic Hearing Loss, Demonstrated by Zebrafish Connexin 30.3 Homologue Model

**DOI:** 10.3390/cells9051291

**Published:** 2020-05-22

**Authors:** Hsuan-An Su, Ting-Wei Lai, Shuan-Yow Li, Tzu-Rong Su, Jiann-Jou Yang, Ching-Chyuan Su

**Affiliations:** 1 Department of Medical Education, Kaohsiung Chang Gung Memorial Hospital, Kaohsiung 807, Taiwan; soshine@hotmail.com.tw; 2 Department of BioMedical Sciences, Chung Shan Medical University, Taichung 402, Taiwan; w010941@gmail.com (T.-W.L.); syl@csmu.edu.tw (S.-Y.L.); 3 Department of Medical Research, Chung Shan Medical University Hospital, Taichung 402, Taiwan; 4 Antai Medical Care Corporation Antai Tian-Sheng Memorial Hospital, Pingtung 928, Taiwan; a085085@mail.tsmh.org.tw; 5 Department of Beauty Science, Meiho University, Pingtung 912, Taiwan

**Keywords:** non-syndromic hearing loss, zebrafish, GJB2, CONNEXIN 26, Connexin 30.3, behavioral assay

## Abstract

Nonsyndromic hearing loss (NSHL) is of great clinical importance, and mutations in the *GJB2* gene and the encoded human CONNEXIN 26 (CX26) protein play important roles in the genetic pathogenesis. The CX26 p.R184Q mutation was shown to be a dominant-negative effect in our previous study. Previously, we also demonstrated that zebrafish Cx30.3 is orthologous to human CX26. In the present study, we established transgenic zebrafish models with mutated Cx30.3 specifically expressed in the supporting cells of zebrafish inner ears driven by the *agr2* promoter, to demonstrate and understand the mechanism by which the human CX26 R.184 mutation causes NSHL. Our results indicated that significant structural changes in the inner ears of transgenic lines with mutations were measured and compared to wild-type zebrafish. Simultaneously, significant alterations of transgenic lines with mutations in swimming behavior were analyzed with the zebrafish behavioral assay. This is the first study to investigate the functional results of the CX26 p.R184Q mutation with in vivo disease models. Our work supports and confirms the pathogenic role of the CX26 p.R184Q mutation in NSHL, with a hypothesized mechanism of altered interaction among amino acids in the connexins.

## 1. Introduction

Hearing loss (HL) is a common sensory disorder caused by a wide range of genetic and environmental factors, and is of great clinical importance in sensory development, language acquisition, and social interaction [1]. Approximately 80% of nonsyndromic hearing loss (NSHL) is attributed to autosomal recessive inheritance, mostly resulting in prelingual and severe HL [2]. The most frequent autosomal recessive genes causing NSHL include *GJB2*, *SLC26A4*, *MYO15A*, and *OTOF* [2].

Gap junctions, which are composed of connexins, help mediate the potassium circulation in the cochlea, to maintain a high potassium level in the endolymph for normal auditory physiology [3,4]. The human CONNEXIN 26 (CX26) and CONNEXIN 30 (CX30) proteins, which are encoded by the *GJB2* and *GJB6* genes, respectively, are the most abundant connexins in cochlear supporting cells [3,5,6,7]. Mutation of CX26 is the major etiology of NSHL, and similar results have also been reported in Taiwanese patients [2,5]. Mutations in human CX26 mostly have an autosomal recessive pattern, but the CX26 p.R184Q missense mutation has been identified in several NSHL populations with a dominant-negative effect [5,8,9,10]. The abnormal accumulation of CX26 p.R184Q protein in the Golgi apparatus was evident in a cellular study [10].

The zebrafish (*Danio rerio*) is a suitable animal model for research on vertebrate inner ear and hearing impairment [11]. In correspondence with the human inner ear, there are two sensory epithelia in each zebrafish otic vesicle (OV), the anterior utricular macula and posterior saccular macula, which perform vestibular and auditory functions, respectively [12]. The zebrafish genome is highly orthologous to the human genome, facilitating our understanding of vertebrate development and genetic pathology [13]. Tao et al. (2010) reported that zebrafish Cx30.3 (Cx30.3) is orthologous to both CX26/Cx26 and CX30/Cx30 in mammals with similar expression patterns [14]. Furthermore, our previous study showed the high similarity of roles between Cx30.3 and CX26 in the inner ear [15]. 

The anterior gradient 2 (Agr2) protein, which is encoded by the *agr2* gene, is a member of the protein disulfide isomerase family [16,17]. Tang et al. discovered that a 100-bp sequence located at the –2.6 to –2.5 kbp region upstream of *agr2* is a promoter driving genetic expression specifically in the supporting cells of sensory patches in zebrafish OVs, and the promoter enables us to investigate the role of Cx30.3 in the OV supporting cells [18]. Therefore, the aims of the present study were to reproduce the orthologous mutation of CX26 in zebrafish OVs with the agr2 promoter and to demonstrate the effects of mutated Cx30.3 in the OV of the inner ear, with functional and behavioral analyses.

## 2. Materials and Methods

### 2.1. Zebrafish Strain and Maintenance

All zebrafish experiments in this study were conducted with AB wild-type strain of Danio rerio. The zebrafish larvae of wild-type AB strain (WT) and transgenic lines were raised in an incubator at 28.5 °C with 10 h-dark and 14-h light circadian cycle. Embryos were cultivated within fresh egg water at 28.5 °C. All methods pertaining to general maintenance, breeding, microscopic observation, genetic methods, histological methods, and molecular methods, were performed according to the Zebrafish Book [18]. All of the protocols in the present study have been reviewed and approved by the Institutional Animal Care and Use Committee (IACUC) of Chung-Shan Medical University Experimental Animal Center (IACUC Approval No.1415).

### 2.2. Cloning of Wild-Type and Mutant Zebrafish Connexin 30.3 Gene

The preparation of zebrafish Cx30.3 wild-type (Cx30.3WT) expression plasmid in pLEGFP-N1 (pLEGFP-N1::Cx30.3WT) has been previously described [15], with the forward and reverse primers containing restriction endonuclease site ends 5′-EcoRI and 3′-BamHI (Table 1). The Cx30.3 mutants were generated by performing oligonucleotide-directed mutagenesis with Stratagene QuikChange Site-Directed Mutagenesis Kit (Stratagene, La Jolla, CA, USA), including Cx30.3 p.R186K (c.557 G>A) and Cx30.3 p.R186Q (c.556 A>C, c.557 G>A). All primer pairs used in the experiments are listed in Table 1. DNA sequences of all constructs, pLEGFP-N1::Cx30.3WT, pLEGFP-N1::Cx30.3R186K and pLEGFP-N1::Cx30.3R186Q, had been confirmed by the restriction digestion and the fluorescent dideoxy-terminator method, with a DNA sequencing kit and an ABI PRISM 3730 (Applied Biosystems Corporation, Waltham, MA, USA).

### 2.3. Expression of Zebrafish Connexins in HeLa Cells

The preparation of human epithelioid cervix carcinoma cells (HeLa; ATCC CCL-2; American Type Culture Collection, Rockville, MD, USA) and the methods of cell transfection have been previously described [15]. After the transfection of pLEGFP-N1 vector containing DNA encoding Cx30.3WT, mutant Cx30.3R186K, or mutant Cx30.3R186Q proteins, the HeLa cells were maintained in the minimal essential Dulbecco’s modified eagle medium (DMEM) with supplements as aforementioned, and used for the subsequent functional analysis.

### 2.4. Immunofluorescence Staining and Fluorescent Labeling on the Endoplasmic Reticulum of Post-Transfection HeLa Cells

A direct fluorescent protein fusion method associated with an in-frame fusion EGFP on the C-terminal of Cx30.3 was used for analyzing the subcellular localization of Cx30.3 in HeLa cells. Cells were seeded on microscope coverglasses and cultured for 24 h to be 80–90% confluent. Cells were permeabilized in phosphate-buffered saline (PBS) with 0.1% NP-40 for 3 min at room temperature, before being fixed with 1:200 dilution of 4% paraformaldehyde (PFA) at room temperature for 20 min. The cells were then incubated overnight with monoclonal primary antibody against pan-Cadherin (mouse anti-CH19, Abcam, Cambridge, UK), at 4 °C. Alexa-Flour 594-conjugated goat anti-mouse IgG (H+L) (Invitrogen, Waltham, MA, USA) with 1:2000 dilution was used as the secondary antibody at room temperature for 3 h. The cells were counterstained with 4’,6-diamidino-2-phenylindole (DAPI) before being mounted in Fluoromount-G (SouthernBiotech, Birmingham, AL, USA). ER-Tracker Blue-White DPY (Invitrogen) was a cell-permeable and ER-selective dye, and was used for incubation at 37 °C for 10 min, with a working concentration of 1 µM, followed by a nuclear counterstaining with 1.25 ng/µL propidium iodide (Invitrogen) staining solution at 37 °C for 3 min. The cells were then fixed with 4% PFA before being mounted in Fluoromount-G (SouthernBiotech). Stained cells were observed under fluorescent lights with wavelength 374nm for excitation, and 430–640 nm for emission. The images were examined and obtained with Axioplan 2 fluorescence microscope imaging system (Zeiss, Jena, Germany).

### 2.5. Generation of Germline Transgenic Zebrafish

A 100bp sequence upstream of zebrafish anterior gradient 2 homologue (agr2) gene has been reported to express specifically in the otic vesicles, and the preparation of plasmid Tol2-exo200-agr2-96ecto-EGFP, associated with a fluorescent protein enhanced green fluorescent protein (EGFP), was kindly provided by Sheng-Ping L. Hwang and colleagues [19]. Multisite gateway cloning technology was applied to establish transgenic zebrafish lines according to manufacturer’s instruction (Invitrogen), and therefore three expression plasmids, pDestTol2pA: agr2-96ecto-Cx30.3WT-EGFP-polyA, pDestTol2pA: agr2-96ecto-Cx30.3R186K-EGFP-polyA, and pDestTol2pA: agr2-96ecto-Cx30.3R186Q-EGFP-polyA, were generated. The Tol2 transposase mRNA was generated by linearization of pCS2+-transposase with NotI digestion, followed by an in vitro transcription reaction with SP6 RNA polymerase (Ambion, Austin, TX, USA). The Tol2 expression plasmids were diluted to 30 ng/µL, mixed with 30 ng/µL transposase mRNA at a molar ratio of 1:1. The mixture of plasmid and mRNA was microinjected into embryos at one-cell stage using a Nanoject II automatic injector (Drummond, Broomall, PA, USA), with each injection containing 2.3 nL of the mixture. The embryos after microinjection were raised to adulthood as the F0 generation. F0 zebrafish were outcrossed with AB wild-type strain to reproduce F1 embryos, the larvae of which could be visualized with fluorescent expression in the inner ear. The EGFP-positive F1 larvae were separately raised to sexual maturity (generally 4 to 6 months), and maintained for incross or outcross to reproduce genetically stable EGFP-positive F2 generation.

### 2.6. Immunohistochemical Staining of Tissue Sections

To highlight the positions of fluorescent proteins and structures of the inner ears, 12dpf zebrafish was selected and fixed with 4% PFA in PBS overnight. After being embedded in 1% agarose, the samples were dehydrated orderly with 70%, 80%, 90%, and 100% ethanol; one hour for each. Deparaffinization was performed with Neo-Clear^®^ (Merck, Kenilworth, NJ, USA). After being immersed within Paraplast Plus (McCormick, Hunt Valley, MD, USA) at 65 °C overnight, the samples were immersed at 65 °C for one hour within a 7:3 mixed paraffin of Paraplast Plus (McCormick) and Paraplast HM (McCormick), respectively. The samples were embedded in the mixed paraffin with AMOS TEC-2800 Embedding Center, and cooled upon AMOS TEC-2800 Cryo Console, before being stored at −20 °C. By AMOS AEM 450, the samples embedded in frozen paraffin were sliced with 5µm-thickness. After deparaffinization with Neo-Clear^®^ (Merck), the samples were rehydrated orderly, with 100%, 95%, 70%, and 0% ethanol 5 min for each, followed by antigen retrieval with Tris-EDTA at 95 °C for 1 h. After immersion with 3% blocking solution for 2 h, the samples were immersed in the primary antibody (1:400) and covered with parafilm at 4 °C overnight. The samples were rinsed with PBST 10 min, 3 times, and then immersed with the secondary antibody (1:400) for 2 h at room temperature. The antibodies and concentrations were listed as follows, 1:400 dilution of mouse anti-GFP monoclonal IgG primary antibody (Santa Cruz Biotechnology, Dallas, TX, USA) to enhance the EGFP fluorescence of the transgenic zebrafish, 1:400 dilution of rabbit pan-Cadherin primary antibody (Invitrogen) as a cell membrane marker, and 1:500 dilution of Alexa Flour 488 goat anti-mouse IgG or Alexa Fluor 594 goat anti-rabbit IgG secondary antibodies (Jackson ImmunoResearch, Ely, Cambridgeshire, UK). A cell-permeable DNA-binding dye with blue fluorescent reactivity, 4’,6-diamidino-2-phenylindole (DAPI, SouthernBiotech) was used for nuclear counterstaining. Finally, the samples were mounted with Neo-Mount^®^ (Merck), before observation under an inverted digital imaging fluorescence microscope (Zeiss Axiovert 200M, Jena, Germany), or a confocal laser scanning Microscope system (Zeiss LSM 510, Jena, Germany).

### 2.7. Measurement of the Structural Alteration in the Inner Ears of Zebrafish

Larvae of 4dpf was embedded with 0.5% agarose in a standardized lateral positioning. The images were obtained under an inverted fluorescence microscope with a 40× magnification, and the maximal distance between the outer edges of the otoliths (otolith distance), anterior otolith diameter, posterior otolith diameter, and distance between the inner edges of the otoliths (otolith space) were measured by using AxioVision Microscopy Software (Zeiss).

### 2.8. Structural Analysis of the Cx30.3 Protein

The SWISS-MODEL protein structure homology-modelling system by Swiss Institute of Bioinformatics (https://swissmodel.expasy.org/) was used for an analysis of the structures of Cx30.3 proteins and its variants [20,21,22,23,24]. The amino acid sequences of the Cx30.3 proteins were extracted from the NCBI database. The structures of the proteins were illustrated and the distances between amino acids of interest were measured. The amino acid sequences of Cx30.3 variants were listed in Supplementary Table S1.

### 2.9. Zebrafish Behavioral Assay

To survey the behavioral alteration of transgenic zebrafish, 10dpf larvae was transferred into 24-well TPP^®^ tissue culture plates individually, with 1 mL egg water for each. After sitting still for 10 min, the larvae were carefully placed into the DanioVision (Noldus, Wageningen, The Netherlands) system and videos were obtained by EthoVision XT (Noldus) video tracking software. The protocol of trials was set up as tapping for 4 times with 5 intervals of 15 s at the beginning, between, and at last. The tapping caused stimuli with sound and vibration, and was set up at level 8 (the highest). The entire video tracking procedures were performed during 3 pm to 5 pm in an isolated quiet room. The video data were transformed into digital results by EthoVision XT (Noldus) video tracking software. Four parameters were calculated and evaluated majorly, including distance moved, velocity, turn angle, and rotation. Distance moved stands for the length of the track of a zebrafish during the whole course of video tracking, with the minimum distance moved being 0.5 cm, while velocity equals distance moved divided by duration of the tracking. Turn angle was defined as the absolute values of average change in angles of directions, regardless of turning clockwise or counterclockwise. One rotation is counted when a subject completes a cumulative turn angle of 90°, with turns in the opposite direction of less than 45° ignored.

### 2.10. Statistical Analysis

All of the digital data were organized by using Microsoft Excel software and were analyzed by using Prism 8 (GraphPad Software, San Diego, CA, USA). One-way ANOVA was mainly performed to examine the differences among parameters. The dispersions of the mean were presented as standard error of the mean (SEM). Statistical significance was considered as *p* < 0.05.

## 3. Results

### 3.1. The Structural Analysis of the Cx30.3 Variants

**Figure 1:**
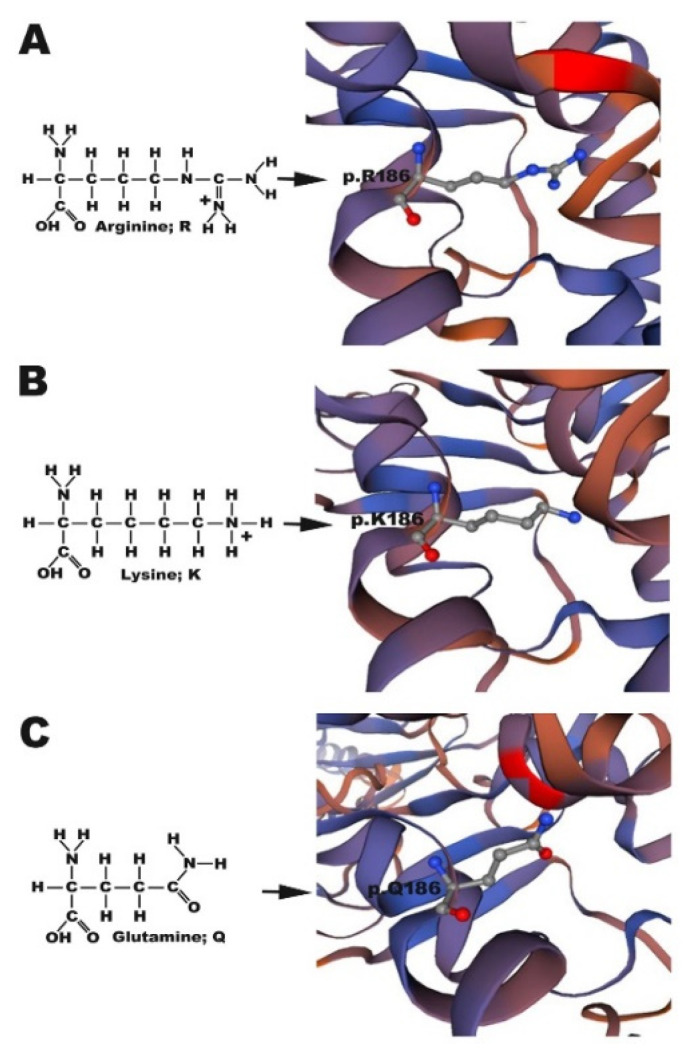
Structure simulation of (**A**) Cx30.3WT p.R186, (**B**) Cx30.3R186K p.K186 and (**C**) Cx30.3R186Q p.Q186. Arginine and lysine have a positively charged side chain to form strong ionic bonds, which is absent in glutamine. The structures of Cx30.3 proteins were simulated by using SWISS-MODEL online software. The amino acid of interest is shown in ball-and-stick style, where red atom represents oxygen, blue atom represents hydrogen, and gray atom represents carbon.

Based on a report by Tao [14], amino acid sequence alignment of human CX26 and zebrafish Cx30.3 indicated that p.R184 in CX26 is homologous to p.R186 in Cx30.3 (Figure S1). However, while GJB2 c.551G>A produced in CX26 p.R184Q in humans, cx30.3 c.557 G>A produced Cx30.3 p.R186K in zebrafish. To simulate the pathologic phenotype of HL, a double-point mutation, cx30.3 c.556 A>C and c.557 G>A, was generated with the site-directed mutagenesis kit to produce the Cx30.3 p.R186Q mutant. The structures of arginine, glutamine, and lysine could be distinguished by their side chains, which are composed of 2 nitrogen molecules, 1 nitrogen and 1 oxygen molecule, and 1 nitrogen molecule, respectively. The structures of Cx30.3 R186R (WT), Cx30.3 R186K, and Cx30.3 R186Q were simulated and illustrated (Figure 1). According to a report by Maeda et al., p.R184 in hCX26 forms hydrogen bonds or salt bridges with p.D46 of the same connexin and with p.E47 and p.Y65 of the adjacent connexin [25]. The distances between Cx30.3 p.R186 of one connexin and adjacent amino acids of the neighboring connexin were measured. The p.R184, p.E47, p.Y65, and p.D46 in CX26 homologously corresponded to p.R186, p.E47, p.Y65, and p.D46 in Cx30.3, respectively. The distances from p.R186 to the other three amino acids were the longest in Cx30.3 R186K, followed by Cx30.3 R186Q and Cx30.3 WT (Table 2).

### 3.2. Expression Pattern of Cx30.3WT, Mutant Cx30.3R186K and Cx30.3R186Q Proteins in HeLa Cell Lines

**Figure 2:**
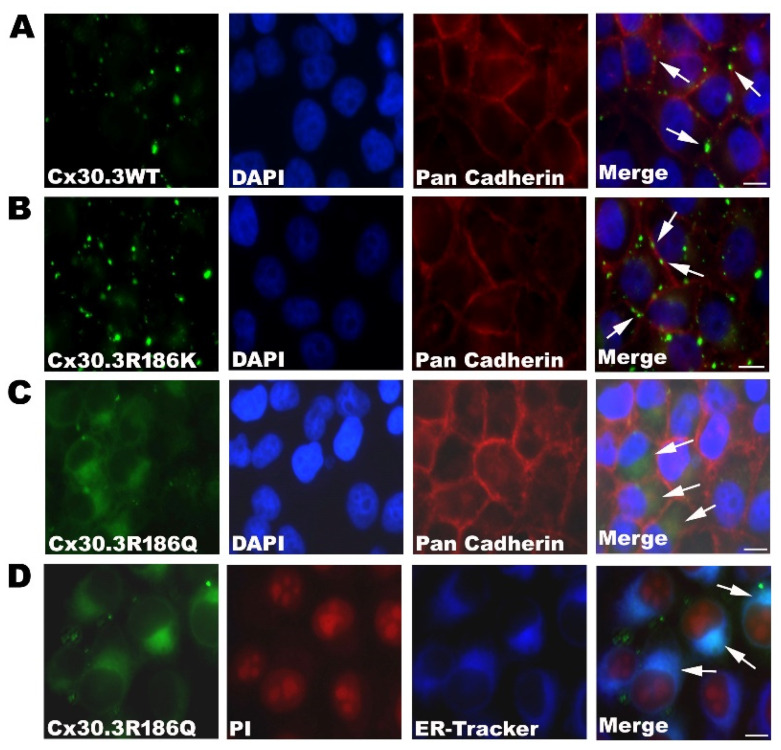
Localization of (**A**) Cx30.3WT, (**B**) Cx30.3R186K, and (**C**) Cx30.3R186Q proteins in HeLa cells. These cells were processed for immunofluorescence analysis using pan-Cadherin antibody (red) and DAPI (blue). The HeLa cells transfected with Cx30.3WT and Cx30.3R186K all showed positive immunofluorescent reactions at the plasma membrane. However, Cx30.3R186Q were found accumulated around the nuclear regions. (**D**) The cells were labeled with 1µM ER-TrakerTM Blue-White DPY, and were analyzed under fluorescence microscopy to identify subcellular localization. The images revealed that Cx30.3R186Q was co-localized at the endoplasmic reticulum. HeLa cells were counterstained with PI (red) to highlight the nuclei. White arrows indicated the cellular localization of Cx30.3 proteins. †Scale bars represent 50 µm.

The constructed plasmids, pLEGFP-N1::Cx30.3WT, pLEGFP-N1::Cx30.3R186K, and pLEGFP-N1::Cx30.3R186Q, were transiently transfected into connexin-deficient HeLa cells with immunofluorescent staining, using primary antibodies against pan-cadherin. The subcellular distribution of the Cx30.3 WT proteins localized to the plasma membrane with the formation of intercellular gap junctions is consistent with our previous research [15]. Similar to Cx30.3WT, EGFP-tagged Cx30.3R186K proteins were localized to the plasma membrane between adjacent cells. However, EGFP-tagged Cx30.3R186Q proteins were presented within the cytoplasm close to the nuclei (Figure 2A–C). To further identify the subcellular distribution of Cx30.3R186Q, HeLa cells were labeled with 1 µM ER-TrackerTM Blue-White DPY, and Cx30.3R186Q was co-localized with the endoplasmic reticulum (Figure 2D).

### 3.3. Establishment of Transgenic Zebrafish Tg(agr2:Cx30.3-EGFP) Lines

**Figure 3:**
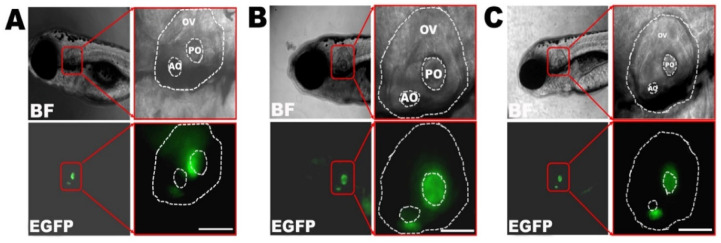
Transgenic zebrafish under inverted fluorescent microscope at 7dpf, including (**A**) Cx30.3WT, (**B**) Cx30.3R186K and (**C**) Cx30.3R186Q transgenic lines under bright field and FITC filter, at 100× and 400× magnification. The green fluorescence highlights the supporting cells within the inner ears. BF, bright field; EGFP, enhanced green fluorescent protein; OV, otic vesicle; AO, anterior otolith; PO, posterior otolith.

The transgenic zebrafish lines of Tg(agr2:Cx30.3WT-EGFP), Tg(agr2:Cx30.3R186K-EGFP), and Tg(agr2:Cx30.3R186Q-EGFP) were established and stabilized until the F2 generation. After microinjection, the inner ears of larvae were visualized with green immunofluorescence at 4 dpf and photographed at 7 dpf. In addition to the autofluorescence presented in the yolks, the green fluorescence was localized to both the anterior and posterior maculae of the inner ears. Green fluorescence encompassing the hair cells with palisading contours could be visualized in the anterior maculae under 400× magnification, featuring the supporting cells (Figure 3). To further ascertain the genotypes of the transgenic fish, genomic DNA was obtained from biopsied fins and was sequenced, which confirmed *cx30.3* c.557 G>A (p.R186K) and *cx30.3* c.556 A>C and c.557 G>A (p.R186Q) mutations (Figure S2).

### 3.4. The Expression Pattern of agr2-Promoted EGFP-Tagged Zebrafish Cx30.3 in Inner Ears 

**Figure 4:**
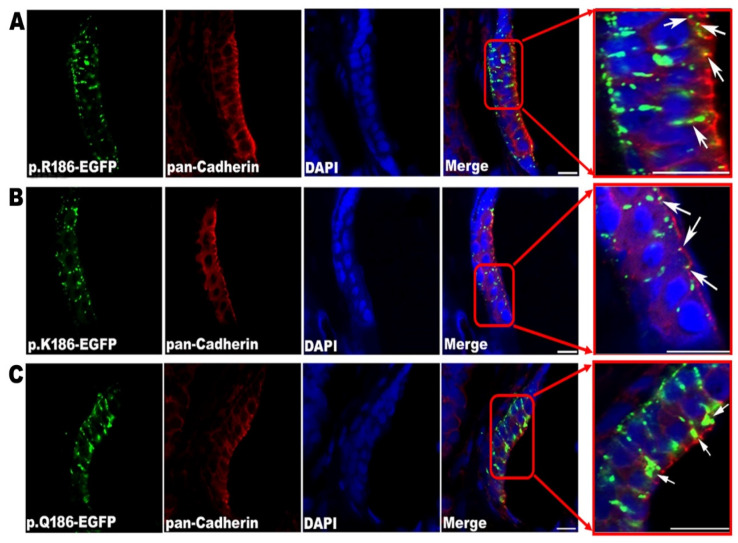
Confirmation of the location of Cx30.3 proteins by immunofluorescent staining, demonstrating the anterior maculae of (**A**) Cx30.3WT, (**B**) Cx30.3R186K and (**C**) Cx30.3R186Q. White arrows point out the green fluorescent Cx30.3 variants. The patterns of Cx30.3WT and Cx30.3R186K proteins were punctiform and spread along the plasma membrane against the adjacent cells, while the Cx30.3R186Q was much more accumulative and concentrated in the cytoplasm. These tissues were processed for immunofluorescence analysis using GFP antibody (green), pan-Cadherin antibody (red) and DAPI (blue). The images were captured with a Zeiss LSM510 confocal laser scanning microscope system at 630× magnification.

To investigate the in vivo presentations of mutant Cx30.3 proteins in adult fish, IHC staining of the frozen sections were visualized with a confocal laser scanning microscope. The Cx30.3WT and Cx30.3R186K variants were punctiform and localized on the plasma membranes against the adjacent cells, while Cx30.3R186Q presented in accumulative patches around the nuclei (Figure 4).

### 3.5. Structural Alteration of the Zebrafish Inner Ear

**Figure 5:**
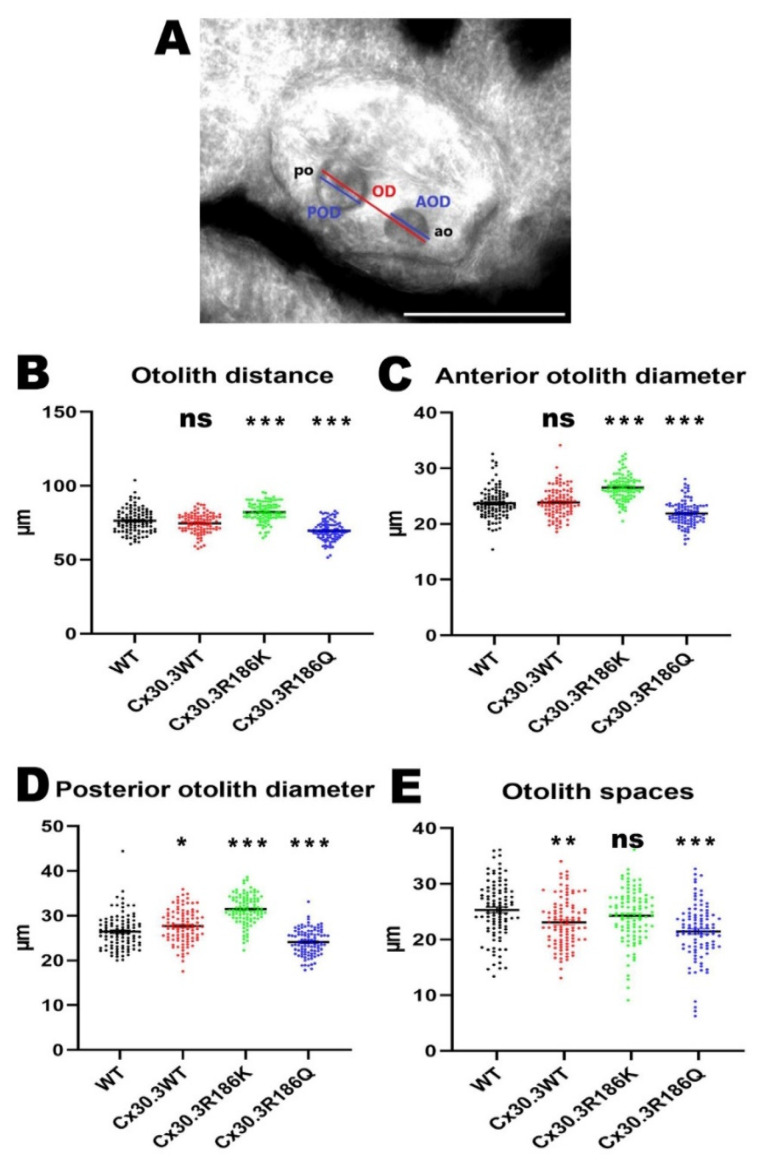
The Cx30.3R186K and Cx30.3R186Q mutations caused structural alterations in transgenic zebrafish compared to WT zebrafish. (**A**) The diagram of the measurement on inner ear structures where the scale bar represents 100µm. Measurement results of (**B**) otolith distances, (**C**) anterior otolith diameters, (**D**) posterior otolith diameters, and (**E**) otolith spaces in WT and transgenic zebrafish. The inner ears were measured by using AxioVision Microscopy Software (Zeiss) at 400× magnification. Values of the bar chart represent means ± SEM with each *n* = 100. Asterisks denote the significant *p*-value < 0.001 compared to wild-type zebrafish. OD, otolith distance; AOD, anterior otolith diameter; POD, posterior otolith diameter; AO, anterior otolith; PO, posterior otolith; ns, not significant.

In our previous study [15], after inhibiting zebrafish cx30.3 by morpholino, shortening of the otolith distance and the diameters of the otoliths had been noted, suggesting the essential role of cx30.3 in inner ear development. Therefore, we want to further understand the impact of these two mutations, Cx30.3R186K and Cx30.3R186Q, on inner ear development. The maximal otolith distances between the outer edges of anterior and posterior otoliths were measured in 100 larvae for each transgenic fish line, as well as WT zebrafish. The otolith distances were 76.39 ± 8.09 µm, 74.66 ± 6.46 µm, 82.24 ± 6.45 µm, and 69.61 ± 6.48 µm in WT, Tg(agr2:Cx30.3WT-EGFP), Tg(agr2:Cx30.3R186K-EGFP), and Tg(agr2:Cx30.3R186Q-EGFP), respectively. The otolith distances were longer in Tg(agr2:Cx30.3 R186K-EGFP) and shorter in Tg(agr2:Cx30.3R186Q-EGFP), while they were insignificantly altered in Tg(agr2:Cx30.3WT-EGFP), compared to those in WT zebrafish. Increased anterior and posterior otolith diameters were observed in Tg(agr2:Cx30.3R186K-EGFP), while decreased anterior and posterior otolith diameters were measured in Tg(agr2:Cx30.3R186Q-EGFP). The otolith spaces only decreased in Tg(agr2:Cx30.3WT-EGFP) and Tg(agr2:Cx30.3R186Q-EGFP) (Figure 5; Table 3).

### 3.6. Zebrafish Behavioral Assay

**Figure 6:**
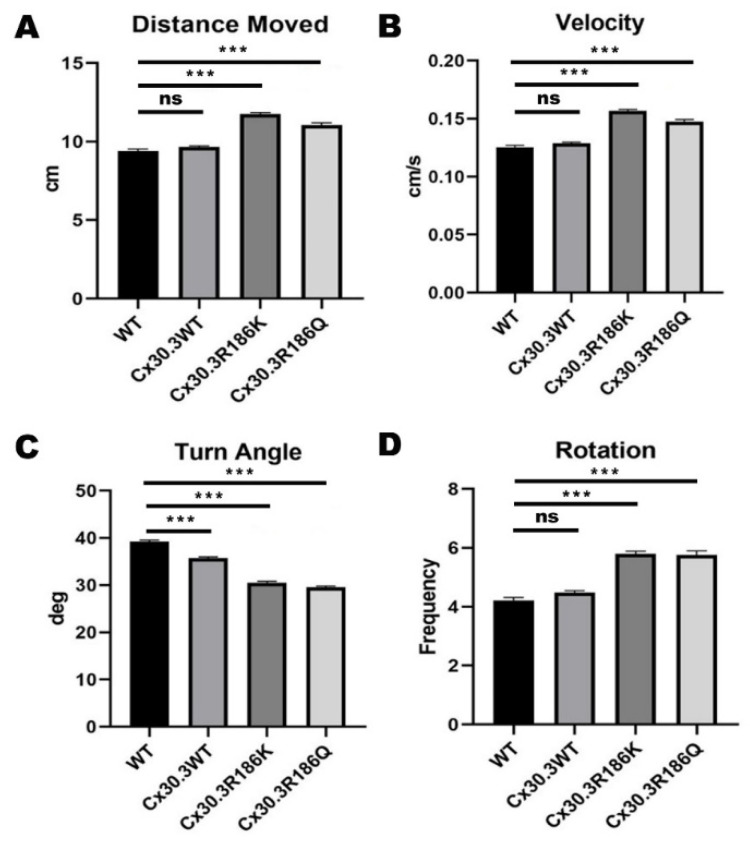
Results of distance moved, rotation, turn angle, and velocity of wild-type AB strain (WT) and transgenic zebrafish by zebrafish behavioral assay, using DanioVision software. (**A**) The distance moved of Tg(agr2:Cx30.3R186K-EGFP) and Tg(agr2:Cx30.3R186Q-EGFP) were significantly more than that of WT and Tg(agr2:Cx30.3WT-EGFP). (**B**) The moving velocities of Tg(agr2:Cx30.3R186K-EGFP) and Tg(agr2:Cx30.3R186Q-EGFP) were significantly higher than that of WT and Tg(agr2:Cx30.3WT-EGFP). (**C**) The turning angles of Tg(agr2:Cx30.3R186K-EGFP) and Tg(agr2:Cx30.3R186Q-EGFP) were significantly lesser than WT and Tg(agr2:Cx30.3WT-EGFP). (**D**) The rotation frequencies of Tg(agr2:Cx30.3R186K-EGFP) and Tg(agr2:Cx30.3R186Q-EGFP) were significantly higher than that of WT and Tg(agr2:Cx30.3WT-EGFP). Values of the bar charts represent means ± SEM with each *n* = 750. Asterisks denote the significant *p*-value <0.001. The denotations of fish lines WT, Tg(agr2:Cx30.3WT-EGFP), Tg(agr2:Cx30.3R186K-EGFP) and Tg(agr2:Cx30.3R186Q-EGFP) were simplified in the figure as WT, Cx30.3WT, Cx30.3R186K, and Cx30.3R186Q, respectively; ns, not significant.

For each genotype of zebrafish, including WT, Tg(agr2:Cx30.3WT-EGFP), Tg(agr2:Cx30.3R186K-EGFP), and Tg(agr2:Cx30.3R186Q-EGFP), 750 larvae were tracked with the EthoVision XT system. The outcomes of interest, including the distance moved, velocity, turn angle, and rotation, were analyzed. For WT, Tg(agr2:Cx30.3WT-EGFP), Tg(agr2:Cx30.3R186K-EGFP), and Tg(agr2:Cx30.3 R186Q-EGFP), the average distances moved were 9.41 ± 0.12 cm, 9.65 ± 0.08 cm, 11.76 ± 0.09 cm, and 11.04 ± 0.15 cm, respectively; the average velocities were 0.125 ± 0.002 cm/s, 0.129 ± 0.001 cm/s, 0.157 ± 0.001 cm/s, and 0.147 ± 0.002 cm/s, respectively; the average turn angles were 39.24 ± 0.30 degrees, 35.76 ± 0.21 degrees, 29.62 ± 0.18 degrees, and 30.47 ± 0.32 degrees, respectively, and the average number of rotations were 4.22 ± 0.10, 4.47 ± 0.07, 5.80 ± 0.08, and 5.76 ± 0.13 rotations, respectively (Figure 6). The transgenic lines with the Cx30.3 variant demonstrated a greater distance moved, faster velocity, and more frequent rotations than wild type and Tg(agr2:Cx30.3WT-EGFP); however, the turn angles were greater in WT and Tg(agr2:Cx30.3WT-EGFP).

## 4. Discussion

The crucial role of connexin genes in the inner ear has been well-established [26,27], and mutations in connexin genes, including *GJB2*, *GJB3* and *GJB6*, have been found in human congenital HL [5,6,28,29,30]. Among the mutations in *GJB2* (CX26), a c.551G>A (p.R184Q) missense mutation, which is located at a highly conserved domain within the second extracellular loop of a connexin protein, has been identified in Ghanaian and Taiwanese populations [5,8,9,31]. An in vitro experiment showed that the p.R184Q missense mutation resulted in the abnormal accumulation of the CX26 protein in the cytoplasm rather than translocation to the plasma membrane [10]. However, the functional effect of the p.R184Q missense mutation has not been demonstrated in vivo. In the present study, we first demonstrated the subcellular location of Cx30.3 R186Q, which is homologous to CX26 R184Q, at the endoplasmic reticulum, other than the plasma membrane. Second, we successfully established zebrafish animal models with Cx30.3 variants, and structural alterations in the OV were observed. Third, we exhibited the altered behavior of transgenic fish with tapping stimuli compared to wild type zebrafish. Furthermore, the structures of the Cx30.3 mutants were simulated, and their functional abnormality was discussed.

The atoms in the side chains of arginine, glutamine, and lysine differ, which likely either weakens or enhances chemical bonding between molecules. As arginine and lysine both possess a positively charged side chain, they tend to form strong ionic bonds with other negatively charged ligands; in contrast, the inability of glutamine to form an ionic bond is likely the key reason for the impaired translocation of Cx30.3 p.R186Q to the plasma membrane. Furthermore, the arginine in the CX26 p.R184P mutation found in the Indian population causing CX26 accumulation in the cytoplasm was replaced by a proline, which is also a nonpolar amino acid [31]. Therefore, as a part of the highly conserved domain at Cx30.3 p.R186 or CX26 p.R184, mutations from arginine to other ionic bond-forming amino acids lead to the intact translocation but impaired functionality of Cx30.3/CX26 at the plasma membrane, while mutations to other nonpolar amino acids cause defective translocation to the plasma membrane. Hydrogen bonds or salt bridges have been described to connect the CX26 p.R184 of one connexin with the p.D46 of the same connexin and with the p.E47 and p.Y65 of the adjacent connexin [25]. The distances from Cx30.3 p.R186K and Cx30.3 p.R186Q to the corresponding bonding atoms were longer than those in Cx30.3 p.R186, which might also result in weaker or compromised bonding, and consequently, abnormal folding or loss of function. 

In zebrafish, the inner ear development follows a similar role to that found in other vertebrates, although some morphological patterns are different. During developmental processes, otolith precursor particles are initially distributed throughout the otic vesicle lumen, and then tethered to the tips of the immotile hair cell kinocilia at the otic vesicle poles, forming two otoliths [32]. Previously our study, we demonstrated its characterization using morpholinos directed against the normal expression of Cx30.3 proteins. The knockdown phenotypes of zebrafish *cx30.3* gene showed significant phenotypic changes in inner ear development, resulting in narrower anterior and posterior otoliths and shorter distances [15]. As shown in our results, abnormal distribution of otoliths, reduced otolith size and shorter distances were demonstrated in CX30.3R186Q mutant with small inner ear phenotype. These results are similar with morpholinos knockdown *cx30.3* gene study. In contrast to the Cx30.3R186Q variant, Cx30.3R186K showed increased otolith size and longer distances in this study. This is very interesting and worthy of further research. The current data could only provide the evidence that Cx30.3 mutations contribute to structural alteration. Whether there is a direct association between structural and behavioral change is not known. By the measurements of structural alteration, we would like to showcase the developmental defect caused by the mutations, to provide implication for future investigation on the interaction between genetic alteration and inner ear development.

In the behavioral analysis, significant findings were found between the Cx30.3 mutant and control groups. The distance moved and velocity showed similar results. Transgenic zebrafish with Cx30.3 p.R186K and Cx30.3 p.R186Q mutations demonstrated a longer distance moved compared to WT and transgenic zebrafish with Cx30.3 WT. With a tapping stimulus, the perception of the zebrafish theoretically decreased because of the mutant *cx30.3* genes, but the decrease in mechanosensory perception might not be phenotypically observable, due to compensation by reserved auditory function as well as lateral line neuromasts. However, these mutations are likely to cause instability during swimming, because the anterior utricular maculae primarily mediate vestibular function in zebrafish [12], consequently resulting in a greater distance moved and higher velocity. The frequency of rotation was significantly higher in Tg(agr2:Cx30.3 R186K-EGFP) and Tg(agr2:Cx30.3 R186Q-EGFP) than in the WT and Tg(agr2:Cx30.3 WT-EGFP); paradoxically, the average turn angles were significantly smaller in Tg(agr2:Cx30.3 R186K-EGFP) and Tg(agr2:Cx30.3 R186Q-EGFP). The findings supported the concept that the transgenic zebrafish with Cx30.3 mutations swam with significant instability because a smaller turn angle with a higher rotation frequency reflected very frequent changes in directions. The Cx30.3 p.R186K and Cx30.3 p.R186Q mutations in the inner ears led to behavioral alteration in transgenic zebrafish, which was probably caused by decreased mechanosensory perception and/or swimming instability.

Despite the fact that Cx30.3 p.R186 corresponds to CX26 p.R184, the nucleotide sequences of the two species are not absolutely identical. As mentioned, *GJB2* c.551G>A translates into CX26 p.R184Q in humans, while *cx30.3* c.557 G>A translates into Cx30.3 p.R186K in zebrafish. Hence, the Cx30.3 p.R186Q mutation in the present study was a phenotypic model of NSHL, while the Cx30.3 p.R186K mutation was merely a reproduction of the mutation at a nucleotide level. According to the results from our in vitro experiments, Cx30.3 p.R186K showed normal translocation to the plasma membrane. However, Tg(agr2:Cx30.3R186K-EGFP) displayed an altered behavioral pattern which was similar to Tg(agr2:Cx30.3R186Q-EGFP) in the behavioral analysis, which significantly differed from WT and Tg(agr2:Cx30.3WT-EGFP), suggesting that Cx30.3 p.R186K might influence the oligomerization or functionality of the gap junction, with normal translocation to the plasma membrane. Although Cx30.3 p.R186K did not correlate to any observed clinical conditions, the behavioral alteration and still supports that the Cx30.3 p.R186 site, or CX26 p.R184, plays an important role in either folding, translocation, oligomerization, or functionality of the protein. 

At present, we know that many diseases may be caused by gene mutations. In addition, different mutations in this gene or different amino acid changes in the same position of the gene will also cause the same disease. In this study, we found that Cx30.3R184Q mutant protein was impaired in trafficking to cell membrane and then consequently concentrated in the cytoplasm, close to the nucleus. The subcellular location of Cx30.3R186Q is homologous to CX26 R184Q. Simultaneously, the abnormal distribution of otoliths, reduced otolith size and shorter distances were demonstrated in CX30.3R186Q mutant, with small inner ear phenotype. These results are similar with morpholinos knockdown cx30.3 gene study [15]. Therefore, CX30.3R186Q mutant may affect the function of inner ear function. In the behavior assay, we also confirm the significant behavioral changes in the transgenetic zebrafish with CX30.3R186Q mutant. In contrast to the Cx30.3R186Q variant, Cx30.3R186K show that membrane trafficking is normal and increased otolith size and longer distances in this study. Although we did not further analyze the functional changes of the Cx30.3R186K mutation in the study, we speculate that the development of this inner ear should be affected from the structural changes of otolith and may result in the function of the inner ear being affected. In this study, we found that the Cx30,3R186K mutant has a similar effect in the behavior assay, but is significantly different with WT or Cx30.3WT (Figure 6). According to these results, Cx30.3 p.R186K and Cx30.3 p.R186Q mutations in the inner ears led to behavioral alteration in transgenic zebrafish, which was probably caused by decreased mechanosensory perception and/or swimming instability.

In the present study, the auditory system of transgenic zebrafish was assumed to be partially compromised, but not totally impaired. The transgenic zebrafish basically possessed endogenous *cx30.3* genes, and the microinjected expression plasmids with transposase introduced additional *cx30.3* mutants into the genome. In addition, the perception of transgenic zebrafish with *cx30.3* mutations might still be sensitive to our tapping stimulation, because neuromasts in the lateral line still serve as mechanosensory organs to mediate a variety of behaviors, including rheotaxis, maintenance of a stationary position in a stream, prey detection, and predator avoidance [33]. Therefore, the transgenic zebrafish with *cx30.3* mutations were able to respond to environmental stimuli, but with observable behavioral alterations.

We acknowledge some limitations of the present study. First, the true effects of Cx30.3 p.R186K and Cx30.3 p.R186Q mutations on protein folding, translocation, and functionality were only hypothesized with indirect observation and rationalization. The abnormal accumulation of Cx30.3 p.R186Q and the altered behavioral presentation of transgenic zebrafish were demonstrated, however, this was without a functional analysis of the mutant proteins, such as a dye transfer assay in the cell line with the Cx30.3 p.R186K mutation where the Cx30.3 formed gap junctions on the plasma membrane with significant behavioral changes. Further functional and structural analyses of the protein are required to better understand the functionality of Cx30.3 p.R186K and the autosomal dominant inheritance of Cx30.3 p.R186Q. The exact mechanism of Cx30.3 variants could be further investigated in future research. Theoretically, Cx30.3 R186K could be transfected into HeLa cells and be examined by dye transfer assay to investigate the functionality of the gap junctions. Second, the mechanosensory perception by lateral lines was not inhibited, due to specific expression in the inner ear by the agr2 promoter and could confound variables in the behavioral analysis. Because the lateral line has a similar role to the maculae in the OV, impaired auditory systems might be compensated by mechanoreceptors in the lateral line. Therefore, the results of the behavioral assay might not fully reflect the extent of auditory impairment. 

## 5. Conclusions

Using a zebrafish animal model with homologous Cx30.3 mutations to CX26, we demonstrated that Cx30.3 p.R186Q and Cx30.3 p.R186K mutations resulted in cellular abnormality, changed the inner ear anatomy, and significantly altered the behavior of the mutants compared to WT and transgenic zebrafish with Cx30.3WT. The pathogenetic role of CX26 p.R184Q in human NSHL was simulated and supported in vivo by our transgenic zebrafish models.

## Figures and Tables

**Table 1:** Oligonucleotide primer pairs for DNA cloning and for site-directed mutagenesis.

Name	Oligonucleotide	T_m_ (°C)
Cx30.3 WT 5′-EcoRI	5′-CgCgAATTCATgAgTTggggAgCACTTTATgC-3′	62.0
Cx30.3 WT 3′-BamHI	5′-CgCggATCCggAACAgTCTTATTgCTCgATgAgTC-3′	
Cx30.3 556A>C-seq	5′-TgACTgTTTCATCTCACAgCCgACAgAgAAgAC-3′	64.3
Cx30.3 556A>C-comp	5′-gTCTTCTCTgTCggCTgTgAgATgAAACAgTCA-3′	
Cx30.3 557G>A-seq	5′-gACTgTTTCATCTCAAAgCCgACAgAgAAgACg-3′	64.33
Cx30.3 557 G>A-comp	5′-CgTCTTCTCTgTCggCTTTgAgATgAAACAgTC-3′	

For DNA cloning, underlined sequences are restriction sites for *EcoRI* and *BamHI*; for site-directed mutagenesis, boldface letters indicate point mutation sites. T_m_, primer melting temperature.

**Table 2:** Measurements of distances between p.R186 of one connexin to another hydrogen bond- or salt bridge-forming amino acids of adjacent or the same connexin in the CX30.3 wild-type and mutant connexins.

	Cx30.3WT	Cx30.3R186K	Cx30.3R186Q
p.R186 to E47 of adjacent connexin (Å)	22.86	23.08	23.00
p.R186 to Y65 of adjacent connexin (Å)	30.26	30.33	30.30
p.R186 to D46 of the same connexin (Å)	8.42	8.51	8.49

**Table 3:** Measurements of inner ear structures in wild-type and transgenic zebrafishes.

	Wild-Type	Tg(agr2:Cx30.3WT-EGFP)		Tg(agr2:Cx30.3R186K-EGFP)		Tg(agr2:Cx30.3R186Q-EGFP)	
	Average	Average	*p*-Value ^†^	Average	*p*-Value ^†^	Average	*p*-Value ^†^
Otolith Distances (µm)	76.39 ± 8.09	74.66 ± 6.46	0.2864	82.24 ± 6.45	<0.0001	69.61 ± 6.48	<0.0001
Anterior otoliths diameter (µm)	23.72 ± 2.80	23.88 ± 2.62	0.9712	26.55 ± 2.24	<0.0001	21.89 ± 2.21	<0.0001
Posterior otoliths diameter (µm)	26.42 ± 3.87	27.68 ± 3.69	0.0489	31.46 ± 3.22	<0.0001	24.11 ± 2.89	<0.0001
Otoliths spaces (µm)	25.31 ± 5.10	23.09 ± 4.39	0.0065	24.29 ± 4.74	0.4347	21.45 ± 5.02	<0.0001

^†^ The *p*-values indicate statistical significance of measurements in transgenic lines compared to those in wild-type zebrafish.

## Supplementary Materials

The following are available online at https://www.mdpi.com/2073-4409/9/5/1291/s1, Figure S1: The alignments of amino acid sequences between human CX26 and zebrafish Cx30.3., Figure S2: Confirmation of the genomic DNA sequences in Tg(agr2:cx30.3-EGFP WT), Tg(agr2:cx30.3-EGFP R186K) with 557G>A, and (C) Tg(agr2:cx30.3-EGFP R186Q) with 556A>C and 557G>A. Table S1: Amino acid sequences of Cx30.3 variants.
