# Supplementary material for: The Functional Role of CONNEXIN 26 Mutation in Nonsyndromic Hearing Loss, Demonstrated by Zebrafish Connexin 30.3 Homologue Model
Source: Cells. 2020 May 22;9(5):1291. doi: 10.3390/cells9051291 (PMC7290585; doi:10.3390/cells9051291)
Supplement: Supplementary file 1 [file cells-09-01291-s001.pdf]

Supplementary Materials

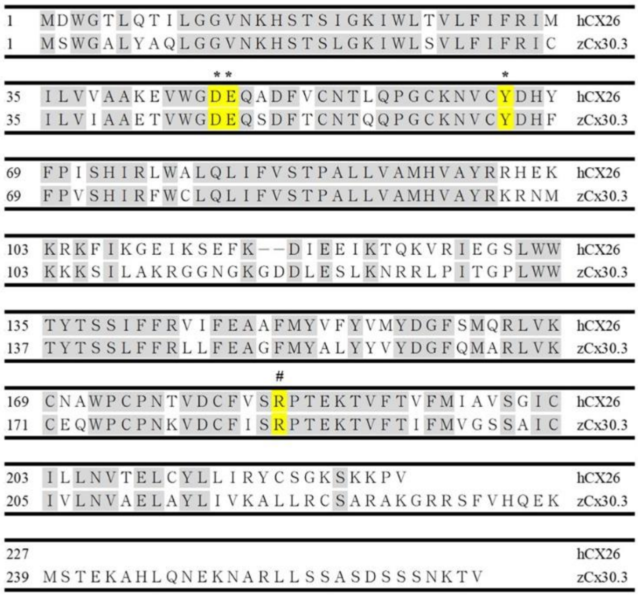

Figure 1. The alignments of amino acid sequences between human CX26 and zebrafish Cx30.3. The gray shading highlights identical residues of the two sequences. The hashtag indicates human p.R184 or zebrafish p.R186, while the asterisks mark p.D46, p.E47, and p.Y65 with bonding connection to p.R184/p.R186 of adjacent or the same connexin. The positions of p.D46, p.E47, and p.Y65 are identical in human and zebrafish.

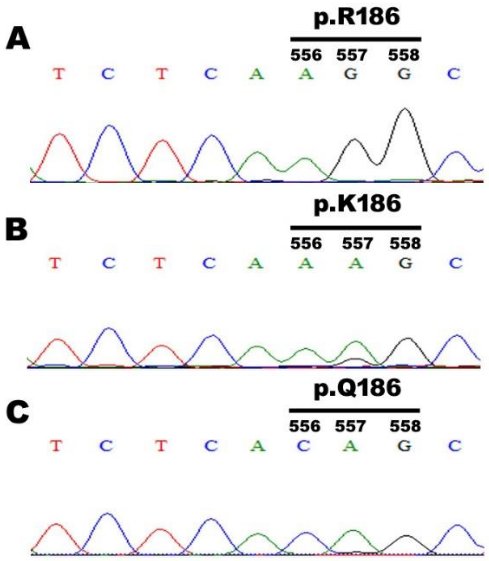

Figure 2. Confirmation of the genomic DNA sequences in (**A**) Tg(agr2:cx30.3-EGFP WT), (**B**) Tg(agr2:cx30.3-EGFP R186K) with 557G>A, and (**C**) Tg(agr2:cx30.3-EGFP R186Q) with 556A>C and 557G>A. The genomic DNA was obtained from the tissues of caudal fins. Polymerase chain reaction was conducted to amplify cx30.3 genes for DNA sequencing.

Table S1. Amino acid sequences of Cx30.3 variants.

Cx30.3 Variants	Amino Acid Sequences
Cx30.3 p.R186	MSWGALYAQLGGVNKHSTSLGKIWLSVLFIFRICILVIAAETVWGDEQSDFTCNTQ QPGCKNVCYDHFFPVSHIRFWCLQLIFVSTPALLVAMHVAYRKRNMKKKSILAKR GGNGKGDDLESLKNRRLPITGPLWWTYTSSLFFRLLFEAGFMYALYYVYDGFQMA RLVKCEQWPCPNKVDCFISRPTEKTVFTIFMVGSSAICIVLNVAELAYLIVKALLRCS ARAKGRRSFVHQEKMSTEKAHLQNEKNARLLSSASDSSSNKTV
Cx30.3 p.Q186	MSWGALYAQLGGVNKHSTSLGKIWLSVLFIFRICILVIAAETVWGDEQSDFTCNTQ QPGCKNVCYDHFFPVSHIRFWCLQLIFVSTPALLVAMHVAYRKRNMKKKSILAKR GGNGKGDDLESLKNRRLPITGPLWWTYTSSLFFRLLFEAGFMYALYYVYDGFQMA RLVKCEQWPCPNKVDCFISQPTEKTVFTIFMVGSSAICIVLNVAELAYLIVKALLRCS ARAKGRRSFVHQEKMSTEKAHLQNEKNARLLSSASDSSSNKTV
Cx30.3 p.K186	MSWGALYAQLGGVNKHSTSLGKIWLSVLFIFRICILVIAAETVWGDEQSDFTCNTQ QPGCKNVCYDHFFPVSHIRFWCLQLIFVSTPALLVAMHVAYRKRNMKKKSILAKR GGNGKGDDLESLKNRRLPITGPLWWTYTSSLFFRLLFEAGFMYALYYVYDGFQMA RLVKCEQWPCPNKVDCFISKPTEKTVFTIFMVGSSAICIVLNVAELAYLIVKALLRCS ARAKGRRSFVHQEKMSTEKAHLQNEKNARLLSSASDSSSNKTV
